# Increased incidence of transient global amnesia during the Covid-19 crisis?

**DOI:** 10.1186/s42466-020-00077-x

**Published:** 2020-09-16

**Authors:** Ralph Werner, Moritz Keller, Johannes C. Woehrle

**Affiliations:** Katholisches Klinikum Koblenz-Montabaur, Department of Neurology & Stroke Unit, Kardinal-Krementz-Str. 1-5, 56073 Koblenz, Germany

**Keywords:** Transient global amnesia, Covid-19, Emotional stress, Takotsubo cardiomyopathy

## Abstract

Since the beginning oft he Covid-19 pandemic we have observed an increased incidence of transient global amnesia, possibly related to emotional stress as a trigger factor.

## Main text

The main clinical feature of transient global amnesia (TGA) is an acute anterograde memory disturbance that resolves within 24 h. It often occurs in the context of physical or emotional stress. Its etiology is still a matter of debate: focal hippocampal ischemia, venous congestion, migraine- or epilepsy-like mechanisms, and metabolic stress have been hypothesized [1].

Since the Covid-19 outbreak in Germany at the end of January 2020 we have noticed an increasing number of patients with TGA in our neurological emergency department in a German academic teaching hospital. Between February 1st and May 15th (when a loosening of the preventive restrictions came into effect because of the decline of new Covid-19 infections in Germany) we diagnosed 16 patients with TGA while the average number of patients in the same period over the last 10 years was 9.7 (SD 2.41, 95%CI 8.3–11.1). One might speculate that the real increase in TGA incidence was even bigger as some patients might have taken distance from presenting to an emergency department in fear of getting infected with SARS-CoV-2 there. Accordingly, many stroke units recently reported lower admission rates of patients with TIA or minor strokes since the beginning of the pandemic [2].

The current TGA patients were slightly older than the more than 200 patients of our single center TGA registry with an average age of 70.8 vs. 66.8 years; 12 (75%) were female compared to 58% in the registry [3]. None of them showed symptoms of Covid-19. Two of the 16 patients suffered their second or third episode of TGA, respectively. One patient, a 86-year-old woman, presented with coincident TGA and Takotsubo cardiomyopathy and was referred to the chest pain unit immediately.

Our hypothesis is that the social distancing during the lockdown, uncertainty concerning the future and not least the fear of getting infected increase the stress level in the community which might trigger TGA [4]. We do not assume that the virus itself causes TGA by an encephalitic autoimmune pathology or direct invasion of the CNS as the clinical features were not different from that of “common” TGA.

For Takotsubo cardiomyopathy, that might share pathophysiological mechanisms with TGA [5, 6], it has been reported that it can occur in the context of natural disasters like e. g. earthquakes [7]; the first cases in the context of Covid-19 have been published recently [8–10].

We are very interested to learn if neurologists in other hospitals have made similar observations.

## Data Availability

The datasets used and/or analysed during the current study are available from the corresponding author on reasonable request.
